# The Synthesis, Characterization, and Protein-Release Properties of Hydrogels Composed of Chitosan-*Zingiber offcinale* Polysaccharide

**DOI:** 10.3390/foods11182747

**Published:** 2022-09-07

**Authors:** Yongshuai Jing, Yameng Zhang, Wenjing Cheng, Mingsong Li, Beibei Hu, Yuguang Zheng, Danshen Zhang, Lanfang Wu

**Affiliations:** 1 College of Chemistry and Pharmaceutical Engineering, Hebei University of Science and Technology, 26 Yuxiang Street, Shijiazhuang 050018, China; 2 College of Pharmacy, Hebei University of Chinese Medicine, 3 Xingyuan Road, Shijiazhuang 050200, China

**Keywords:** *Zingiber offtcinale*, polysaccharide, chitosan, hydrogels, drug release, degradation

## Abstract

Most proteins given orally have low bioavailability and are easily eliminated by rapid metabolism in vivo. In order to immobilize the drug at the site of administration and delay its release, a natural, gentle release system was designed. In this study, a heteropolysaccharide (ZOP) was isolated from *Zingiber officinale* using an ultrasonic assisted extraction method. ZOP Ara = 1.97: 1.15: 94.33: 1.48: 1.07. The ZOP/Chitosan (CS) composite hydrogel was synthesized using epichlorohydrin (ECH) as a cross-linking agent. The structure, morphology, and water-holding capacity of the composite hydrogel were characterized. The data showed that the addition of ZOP improved the hardness and water-holding capacity of the material. A swelling ratio test showed that the prepared hydrogel was sensitive to pH and ionic strength. In addition, the degradation rate of the hydrogel in a phosphate-buffered saline (PBS) solution with a pH value of 1.2 was higher than that in PBS with pH value of 7.4. Similarly, the release kinetics of Bovine serum albumin (BSA) showed higher release in an acidic system by the hydrogel composed of ZOP/CS. The hydrogel prepared by this study provided a good microenvironment for protein delivery. In summary, this composite polysaccharide hydrogel is a promising protein-drug-delivery material.

## 1. Introduction

Hydrogels are three-dimensional networks formed by hydrophilic polymers through physical or chemical crosslinking [1]. Because the hydrogel network contains a large number of hydrophilic functional groups, the hydrogel can absorb and retain water in large quantities without destroying its original three-dimensional structure [2]. In recent years, polysaccharide-based hydrogels have emerged as promising biomaterials because of their high water retention and excellent biodegradability and safety [3,4]. The practical applications of the single polysaccharide gel system are not extensive. In order to meet the characteristics, structure, or properties of a specific purpose, polysaccharides can be combined with another synthetic polymer, natural polymer, or inorganic compound to produce a new structure, and at the same time have the advantages of two components, thereby greatly expanding the application scope of the resulting composite gel [5].

*Zingiber officinale* is a zingiberaceae plant [6]. In recent decades, *Z*. *officinale* has attracted much attention due to its multiple biological activities [7]. ZOP is an important component of *Z. officinale*, and has antioxidant [8,9,10], anti-inflammatory, and bacteriostatic properties [6,11] and anti-fatigue activity [12]. In addition, ZOP has good biocompatibility and biodegradability. However, the application of ZOP in hydrogel synthesis is rarely reported. CS is the only cationic polysaccharide in nature [13], which is non-toxic and easy to degrade. It is often used to prepare materials such as hydrogels [14].

Composite hydrogels have attracted more and more attention due to their excellent mechanical properties and wide range of application [15,16]. In this study, mixed hydrogels containing ZOP and CS were prepared and characterized to improve the swelling performance and practicality of hydrogels. The sustained release abilities of BSA were initially explored to confirm whether the developed hydrogel had the properties of a promising sustained-release oral drug.

## 2. Materials and Methods

### 2.1. Experimental Materials and Reagents

*Zingiber officinale* was obtained from Anguo Yaoyuan Trading Co., Ltd. (Anguo, China). The sample was identified as the rhizome of *Zingiber officinale* Roscoe by associate professor Lanfang Wu (Department of Pharmacy, Hebei University of Chinese Medicine). A voucher specimen was deposited at the College of Chemistry and Pharmaceutical Engineering, Hebei University of Science and Technology, China. Chitosan, Standard monosaccharides, T-series dextrans, and 1-phenyl-3-methyl-5-pyrazolone (PMP) were purchased from Aladdin Biochemical Technology Co., Ltd. (Shanghai, China). All other chemicals and reagents used in the experiments were of analytical grade.

### 2.2. Sample Preparation

#### 2.2.1. Extraction of ZOP

The rhizome of *Zingiber officinale* was pulverized in order to obtain the powder. The powder (100 g) was pretreated with 95% ethanol (1:3, *w*/*v*) for 2 h on 2 consecutive occasions to remove pigments, fats, and other alcohol-soluble substances. After 20 min of ultrasound assistance, the dry residues were extracted twice by distilled water (1:25, *w*/*v*) reflux extraction, for two hours each time. The two aqueous extracts were combined, concentrated, and precipitated with 95% ethanol for 12 h. The extract was filtered and freeze-dried to obtain the final sample.

#### 2.2.2. Analysis of the Average Molecular Weight (Mw) and Monosaccharide Composition of ZOP

The homogeneous distribution and average Mw of ZOP were identified by high-performance gel permeation chromatography (HPGPC) [17]. The monosaccharide composition of ZOP was determined according to the reference [18].

#### 2.2.3. Atomic Force Microscope (AFM) Observation

A 5 μg/mL ZOP sample was dropped on a silicon wafer (the silicon wafer immersed in ethanol was vibrated by ultrasonic vibration for 2.5 h, and the alcohol on the surface was wiped), applied evenly and left to dry naturally. Scanning and observation of ZOP was undertaken using the tapping mode.

#### 2.2.4. Preparation of Hydrogels

**Figure 1:**
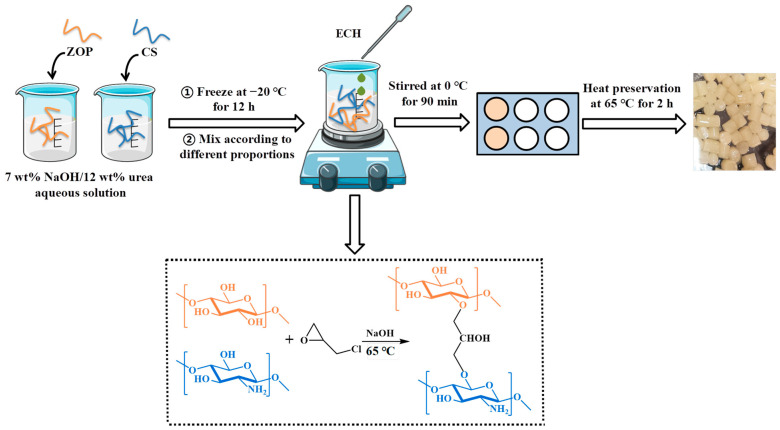
Hydrogel preparation process.

When preparing the hydrogel, we referred to the method described by Chen et al. [19] First, 3 g of ZOP and 3 g of CS were dispersed into 47 g of 7 wt% NaOH/12 wt% urea aqueous solution, respectively. They were fully frozen and then thawed, and stirred evenly until a homogeneous solution was obtained. Different mass ratios of ZOP/CS (ZOP100 (100:00), ZOP70 (70:30), ZOP50 (50:50), ZOP30 (30:70) and ZOP0 (00:100)) were mixed. ECH was used as a crosslinking agent and mixed with polysaccharide solution in a ratio of 1/10 (*v*/*w*), and stirred at 0 °C for 90 min. Then, the polysaccharide mixture was kept at 65 °C for 2 h for molding (Figure 1). Finally, it was soaked in deionized water for 3 days to remove residual urea, NaOH, and ECH. 

### 2.3. Characterization of ZOP/CS Hydrogel

#### 2.3.1. Rheological Measurements

Rheometer (HAAKE MARS40, Bruker, Karlsruhe, Germany) was used to measure the rheological properties of five samples. A cylindrical sample with a diameter of 1 cm and a length of 3 cm was cut for a dynamic oscillation test. At 25 °C, the changes in the storage modulus (G’) and loss modulus (G″) were recorded within the range of 1~100 rad/s. The power law model was used to analyze the relationship between G’ and angular frequency (ω) [20]. The formula was as follows:

G’ = k (ω)*^n^* (1)

where G’ is the storage modulus, ω is the angular frequency, and “k” and “*n*” are constant values.

#### 2.3.2. Gel Hardness and Springiness

The experimental conditions of texture analysis (TA-XTplus, US-Stable Micro Systems) refer to the method of Hurler et al. [21] The hydrogel sample was trimmed into a cylinder with a diameter of about 1 cm and a height of about 3 cm. A cylindrical stainless-steel P/5 probe was used for one compression with a force of 5 g. The compression amount was 30% of the original height of the sample. The speed before, during, and after compression was 2 mm/s. The texture analysis test was repeated 3 times for each sample. TPA parameters included hardness and springiness.

#### 2.3.3. FT-IR Spectrometric

Gel powder (1 mg) and 100 mg KBr powder were mixed, ground evenly, and scanned in the frequency range of 4000 to 400 cm^−1^ using an FT-IR spectrophotometer (NEXUS-760, Thermo Nicolet Corp., Madison, WI, USA).

#### 2.3.4. XRD Analysis

The XRD method was as follows: Cu target, tube flow 20 mA, tube pressure 40 kV, λ = 0.154 nm, scanning angle range 10~90°.

#### 2.3.5. TGA Analysis

The thermal stabilities of ZOP100, ZOP70, ZOP50, ZOP30, and ZOP0 were determined using a thermogravimetric analyzer (TA instruments Ltd., Q600, Reston, VA, USA) under nitrogen shielding gas at a heating rate of 10 °C/min at 20 °C~800 °C.

#### 2.3.6. SEM Analysis

The flat section of the hydrogel was selected, then the five samples were fixed with a conductive adhesive and, after it had been sprayed gold, the section of hydrogel was observed under the working voltage of 10 kV.

#### 2.3.7. Water Content of Hydrogels

The determination of moisture content was based on the method described previously [22]. On this basis, it was modified. The quality of the hydrogel was measured before and after freeze-drying and expressed as follows:

(2) $$\mathrm{Water}\;\mathrm{content}\;\left(\%\right)=\frac{W_{0}-W_{1}}{W_{0}}\times 100$$

where W_0_ is the mass of hydrogel before freeze-drying and W_1_ is the mass of hydrogel after freeze-drying (g).

### 2.4. Swelling Behavior

The freeze-dried hydrogels of different proportions were soaked in distilled water until the swelling equilibrium was reached. The swelling rates of the five hydrogels were calculated as follows:

(3) $$\mathrm{Swelling}\;\mathrm{ratio}\;\left(g/g\right)=\frac{M_{s}-M_{0}}{M_{0}}\times 100\%$$

where Ms (g) is the mass of the hydrogel after water absorption and M_0_ (g) is the mass of the hydrogel before water absorption.

According to the same method, the swelling rates of the five hydrogels at different NaCl and pH concentrations were explored.

### 2.5. In Vitro Degradation

In vitro degradation of the hydrogels was studied as previously described [4]. In short, the lyophilized hydrogel samples (100 mg) were immersed in PBS solutions at pH values of 7.4 and 1.2, and the entire system was placed in a 37 °C shaking table and gently shaken (∼100 rpm). The samples were taken out at a predetermined time point, freeze-dried, and weighed. The degradation degree in vitro was calculated as follows:

(4) $$\mathrm{Remaining}\;\mathrm{weight}\;\mathrm{ratio}\;\left(\%\right)=\frac{M_{t}}{M_{i}}\times 100$$

where M_i_ and M_t_ are the initial weight of hydrogel and the remaining dry weight of hydrogel after degradation, respectively.

### 2.6. In Vitro Release

The lyophilized hydrogel was immersed in 0.5 mg/mL BSA aqueous solution (25 mL) for 24 h to load the protein. The loading amount (m_L_) was calculated as follows:

(5) $$\;m_{L}=V_{0}c_{0}-V_{1}c_{1}$$

where V_0_ is the volume of BSA solution (25 mL), c_0_ is the initial concentration of the BSA solution (0.5 mg/mL), V_1_ is the volume of the BSA solution after 24 h, and c_1_ is the concentration of the BSA solution after 24 h.

The lyophilized protein carrier gel was transferred to a PBS solution containing 25 mL of a pH of 1.2 or 7.4. PBS solutions with different pH values were prepared with mixed phosphate, HCl (0.2 M), and NaOH (0.2 M). Then, 1 mL BSA release medium was periodically removed and 1 mL of the fresh medium was added to maintain volume. The cumulative release rate (Er%) of BSA was calculated as follows [22]:

(6) $$\mathrm{Er}\;\%\;=\frac{V_{0}C_{n}+V_{d}\sum_{1}^{n-1}C_{i}}{m_{L}}\times 100$$

where V_0_ and V_d_ are the volume of the original BSA solution (25 mL) and the volume of the removed BSA solution (1 mL), respectively. C_n_ represents the solution’s concentration at different time intervals. m_L_ is the loading amount of BSA.

### 2.7. Determination of Phagocytic Activity

A Cell Counting Kit-8 (CCK-8) was used to test the effect of hydrogels on the cell viability of RAW264.7 cells [23]. RAW 264.7 cells were inoculated in 96-well plates for 24 h. Hydrogel powder (10, 100, 150, 250, and 500 μg/mL) and positive control lipopolysaccharides (LPS) (1 μg/mL) were added into the plates. After incubation in an incubator at 37 °C and 5% CO_2_ for 48 h, the liquid in the 96-well plates was discarded, and 200 μL CCK-8 solution was added to each well. The absorbance at 450 nm was measured with a microplate reader.

### 2.8. Statistical Analysis

All experimental data are shown as mean ± SD. One-way analysis of variance (ANOVA) plus Duncan’s post hoc test (SPSS 26.0, SPSS Inc., Chicago, IL, USA) were used to evaluate the statistical significance. For all results among the different groups, *p* < 0.05 was considered a significant difference.

## 3. Results and Analysis

### 3.1. Extraction of ZOP

Crude polysaccharides (8.61 g) were obtained from the *Zingiber offtcinale*. The extraction rate was 8.61%.

### 3.2. Analysis of the Average Mw and Monosaccharide Composition of ZOP

The molecular weight of two different components of ZOP were 6.04 × 10^6^ Da (7.17%) and 5.42 × 10^3^ Da (92.83%). The monosaccharide composition and molar ratio of ZOP were GlcA: GalA: Glc: Gal: Ara = 1.97: 1.15: 94.33: 1.48: 1.07.

### 3.3. AFM Observation of ZOP

**Figure 2:**
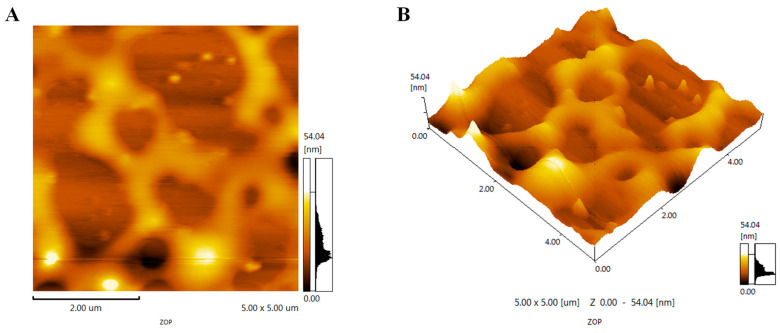
Plan view (**A**) and stereoscopic view (**B**) of AFM.

ZOP was spread on the surface of the mica sheet to form a continuous network structure (Figure 2A), which might be due to the large number of hydroxyl groups in the polysaccharide, or the strong intermolecular hydrogen bond association and tight polymerization of the polysaccharide. The maximum diameter of ZOP was 54.04 nm. A complete net could be clearly seen on the stereogram (Figure 2B), and a surface structure with shallow pits could be seen.

### 3.4. Dynamic Rheological Measurements

**Figure 3:**
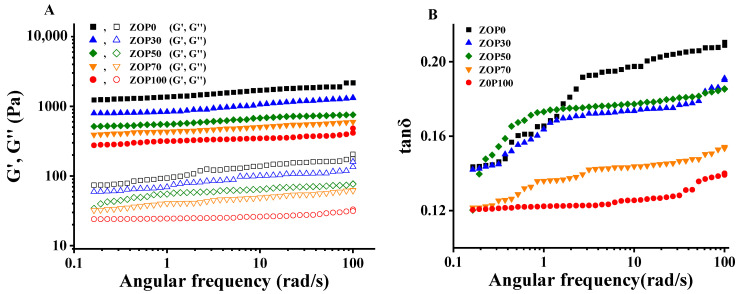
The storage modulus (G′) and loss modulus (G′′) curves of five gels, and the curves of tan δ of five gels changing with angular frequency.

The G′ and G′′ of the five gel samples showed the same increasing trend with the increase in the angular frequency (Figure 3A). Within an angular frequency range of 1~100 rad/s, the G′ of the five gels was correspondingly greater than the G′′, illustrating that the five hydrogels showed primarily elastic solid behavior. The tan δ (G′′/G′) values of the five gels were greater than 0.1 (Figure 3B), indicating that all of the gels were weak gels [24]. The G′ and G′′ of the gel with ZOP added showed a decreasing trend, indicating that the addition of ZOP reduces the viscoelasticity of the CS hydrogel [25]. The rheological parameters of the sample, fitted by a power-law equation, are shown in Table 1. The R^2^ values of the five hydrogels were greater than 0.99, indicating that the results of the hydrogels conformed to the power-law model. The content of ZOP significantly affected the value of *n*, which indicates that ZOP was frequency dependent [26]. In addition, the k value of the hydrogel was decreased by ZOP, which might be an effect caused by the effective volume fraction of the dispersed phase [27].

### 3.5. Gel Properties

Table 1 shows the gel hardness and springiness of the five mixed samples. Adding different proportions of ZOP could significantly affect the textural properties of CS hydrogels. The higher the proportion of ZOP, the greater the hardness and the lower the springiness of the gel. The increase in gel hardness was mainly due to the interaction between ZOP and the CS molecules, which promotes CS aggregation and rearrangement, resulting in the gel becoming harder [20]. Springiness refers to the ability of the gel to return to its original length after being compressed. The pore structure of the gel might be related to its springiness [28,29]. The addition of ZOP filled the voids of the gel, which might prevent the mixed gel from returning to its original length, resulting in a decrease in springiness [29].

### 3.6. FT-IR Spectrometric

**Figure 4:**
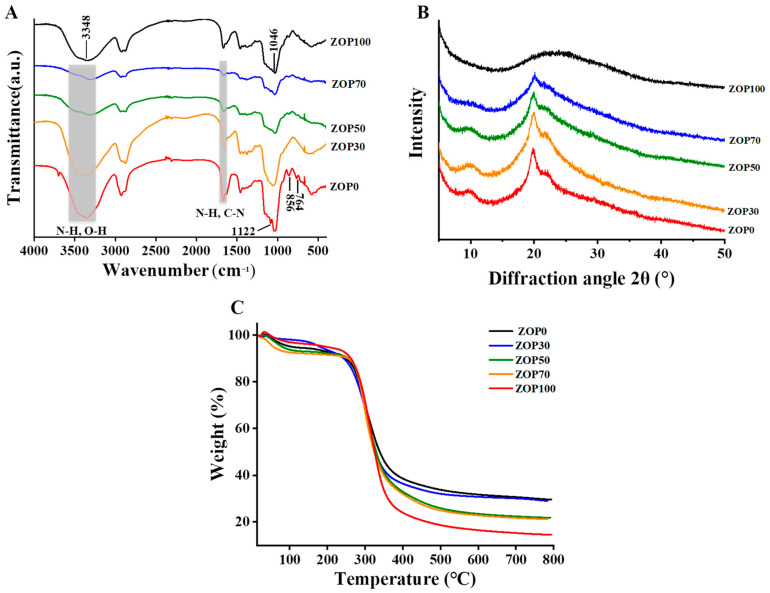
FT-IR spectra of different hydrogels (**A**); XRD of different hydrogels (**B**); TGA of different hydrogels (**C**).

In this paper, FT-IR was used to explore the functional groups of composite hydrogels, and to obtain molecular state information for polymers [30]. The FT-IR spectra of five hydrogels (ZOP0, ZOP30, ZOP50, ZOP70, ZOP100) are shown in Figure 4A. In the FT-IR spectra of the pure CS hydrogel, the wide strong peak at 3200 cm^−1^~3500 cm^−1^ is the multiple absorption band of O–H and N–H overlapping stretching vibrations, and the peak near 1665 cm^−1^ is the flexural vibration of N–H and the stretching vibration of C–N. Chitosan contains a large number of −OH and −NH_2_ groups [19]. In addition, the weak peak at 1122 cm^−1^ belongs to carboxylic ester, while the absorption peaks at 855 cm^−1^ and 765 cm^−1^ might be related to the sulfate group (SO) [31]. In the ZOP spectrum, the wide peak at 3348 cm^−1^ can be attributed to the −OH stretching vibration. The peak at 1048 cm^−1^ represents the C–O stretching vibration in the glucopyranose ring. After adding ZOP to CS hydrogel, the O–H band became weak and blue shifted, which was due to the destruction of hydrogen bonds and the formation of new chemical bonds. These results show that the cross-linking between ZOP and CS was successful.

### 3.7. XRD Analysis

In the XRD pattern, 2θ = 10.9° and 2θ = 20.5° were designated as the crystallization regions of chitosan [32,33,34], while the crystallinity of ZOP was weak. Therefore, in the XRD spectrum of the mixed hydrogel with added ZOP, the intensity of the two peaks decreased and the peak at 2θ = 10.9° gradually disappeared, as the crystallinity decreased (Figure 4B). This indicates that the intramolecular and intermolecular hydrogen bonds weaken or disappear after the two polysaccharides penetrate each other.

### 3.8. Thermal Properties of the Composed Hydrogels

TGA was used to study the thermal stability of the hydrogels. The thermal stability of the five samples is shown in Figure 4C. From the TGA curve, it can be seen that the five samples have three obvious weight-loss stages. The first stage occurs between room temperature and 250 °C, mainly due to the disappearance of free water. With the increase in temperature, the mass decreases rapidly between 250~400 °C, mainly due to the thermal decomposition of the hydrogels. Based on previous studies, the temperature range of 250~400 °C is the range for polysaccharide degradation [35]. In addition, the thermal decomposition peaks of ZOP100, ZOP70, ZOP50, ZOP30, and ZOP0 were at 313.2 °C, 311.2 °C, 310.8 °C, 309.3 °C, and 306.6 °C, respectively. When the temperature rose to 800 °C, the final mass of ZOP100, ZOP70, ZOP50, ZOP30, and ZOP0 was 14.65%, 21.19%, 21.93%, 29.02%, and 29.66% of the initial mass, respectively. With the increase in ZOP content in composite hydrogels, the degradation degree of the hydrogels increased, which might be related to the biodegradability and thermal degradation characteristics of ZOP [36]. This high temperature degradation shows that the mixture was almost cross-linked and very stable over a wide temperature range [37].

### 3.9. Morphology

**Figure 5:**
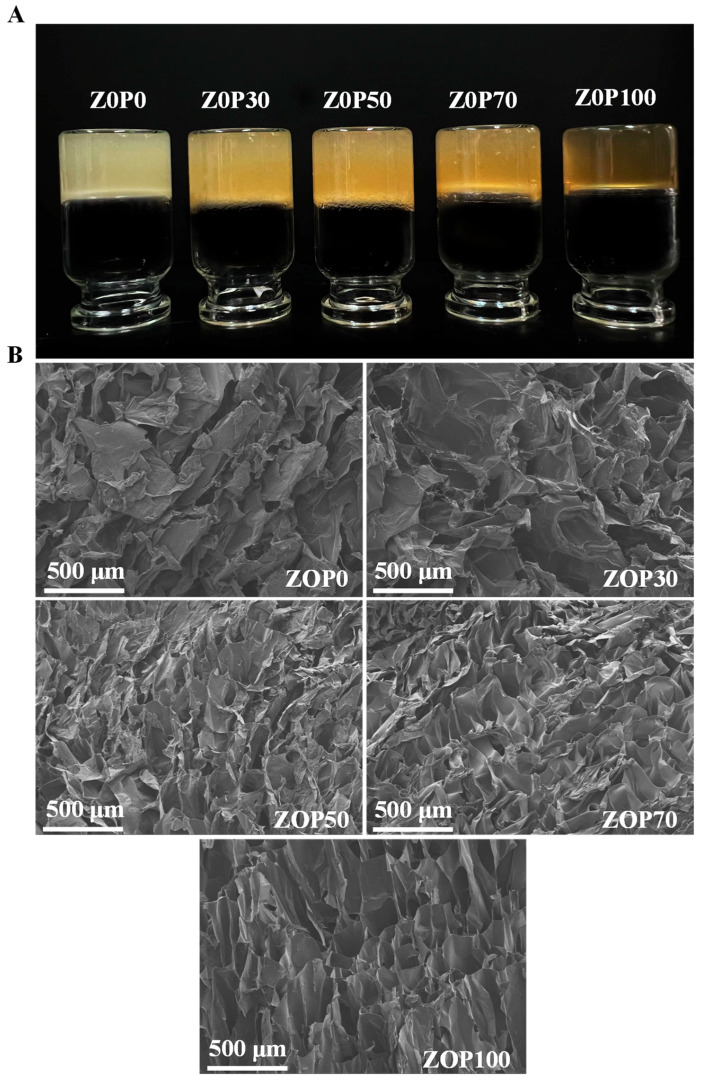
Photographs of different prepared hydrogels (**A**); SEM images of the cross-sectional morphology of the freeze-dried hydrogels (**B**).

Five hydrogels formed under different mixing ratios are presented in Figure 5A. ZOP0 was translucent, milky white, and fragile. On the contrary, ZOP100 was translucent, yellow, and soft. The morphology of the five freeze-dried hydrogels is shown in Figure 5B. SEM images of the five hydrogels showed rough and irregular surfaces and a large number of voids, which indicate a highly porous structure. These porous structures were formed by the disappearance of water molecules during freeze-drying [38]. With the addition of ZOP, these porous structures were arranged more regularly and connected more closely. The arrangement and connection of the pore structures might be related to their swelling capacity. The ZOP100, with tight connections and a regular pore structure, had a higher swelling rate.

### 3.10. Water Content of the Hydrogels

**Figure 6:**
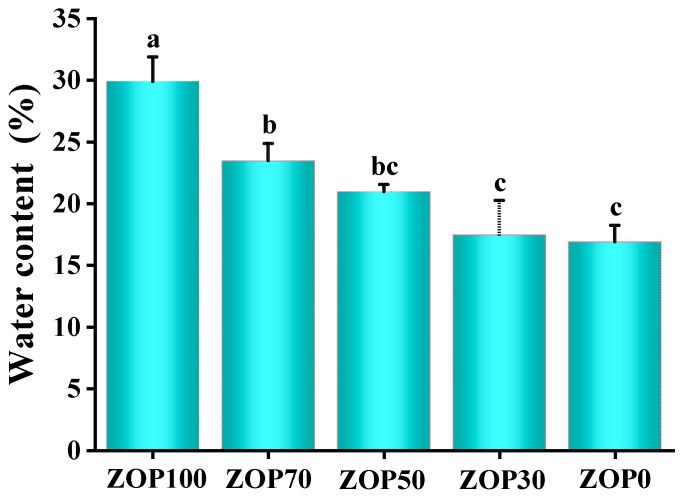
Water-holding capacity of different hydrogels. Note: different letters indicate significant differences in mean values (*p* < 0.05).

The moisture content of the hydrogels was an important criterion to measure their usability [39]. As shown in Figure 6, after adding ZOP to CS, the water content of the hydrogels increased significantly, from 16.89 ± 1.34% of ZOP0 to 29.9 ± 1.99% of ZOP100. According to previous studies, this might be due to the high hydrophilicity, high water-holding capacity, and high sustainability of natural plant polysaccharides, thereby improving the water content of pure CS hydrogels [40]. Therefore, the addition of ZOP increased the water content of the hydrogels and could embed more hydrophilic functional groups and bioactive substances into the hydrogel network [41,42].

### 3.11. Swelling Behavior

**Figure 7:**
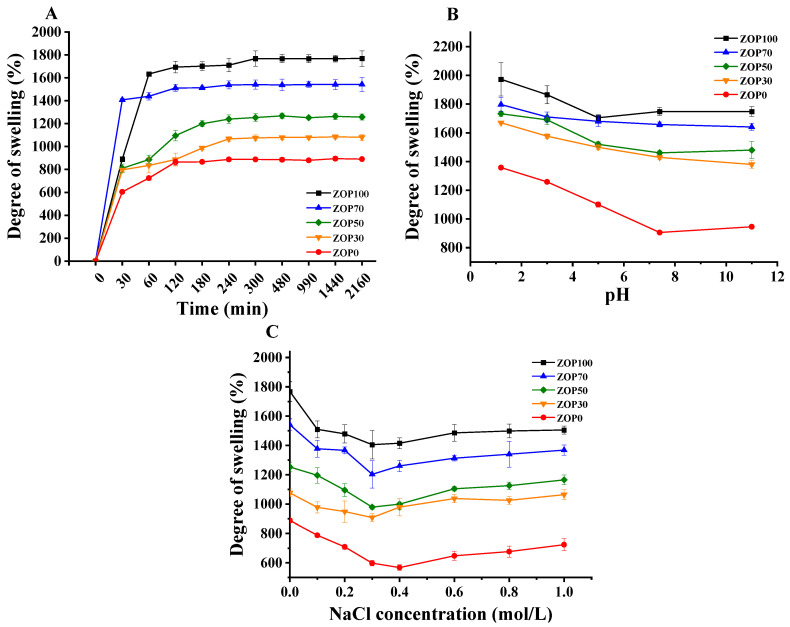
Swelling behavior of the different hydrogels: swelling kinetic curves in deionized water at 25 °C (**A**); swelling ratios values in PBS with pH values ranging from 1.2~7.4 at 25 °C (**B**); swelling ratio values in NaCl solutions with concentrations from 0.01~1 M at 25 °C (**C**).

The swelling properties of hydrogels are closely related to their further applications [43]. Figure 7 shows the swelling rates of five hydrogels at different time, pH value and ionic strength. As shown in Figure 7A, in distilled water at 25 °C, the swelling rate of the hydrogel increased with time. Different proportions of hydrogels reached equilibrium after 5 h. In this study, the SR of the ZOP100 hydrogel was the highest, at about 1769 ± 34.48% after 20 h; that of the ZOP0 hydrogel was the lowest, while that of h was only 887 ± 14.24% after 20 h. It is worth noting that SR and the additional amount of ZOP showed the same trend. In this study, ZOP penetrated into the CS network and improved the interaction between hydrogels and water molecules by increasing the content of hydrophilic groups, such as hydroxyl, so as to enhanced the hydrophilicity of composite hydrogels [44]. Studies have shown that the porosity of hydrogels is closely related to the swelling rate of ZOP/CS-based hydrogels. These pores allow the direct penetration of water, accelerate the diffusion of water fluid in the polymer network, and increase the swelling rate of the hydrogel [45]. 

As shown in Figure 7B, the pH value of the solution could significantly affect the absorption process. With the increase in pH, the swelling rate of the hydrogels decreased. This is because, at a low pH [46], there is protonation of chitosan in hydrogels (−NH_2_ is converted to −NH_3_^+^), which enhances the interaction between polysaccharide chains, so the swelling rate of hydrogels increases. In the PBS solution with a pH of 7.4, the amino deprotonation of CS resulted in the decrease of the SR of the hydrogel [47].

Different concentrations of NaCl were used to evaluate the ion sensitivity of ZOP/CS hydrogels. Figure 7C shows that, when the NaCl concentration increases from 0 mol/L to 0.3 mol/L, the SR of hydrogel shows a downward trend. This is because the ion concentration in the environment where the hydrogel was located is too high, and a higher ion concentration would generate electrostatic repulsion with polycations, resulting in a decrease in the osmotic pressure difference between the inside and outside of the hydrogel, making it difficult for the solvent to enter the hydrogel network [48]. In addition, adding ZOP to CS hydrogels could increase SR. Therefore, it can be deduced that the ZOP/CS hydrogel was sensitive to ionic strength. 

### 3.12. In Vitro Degradation

**Figure 8:**
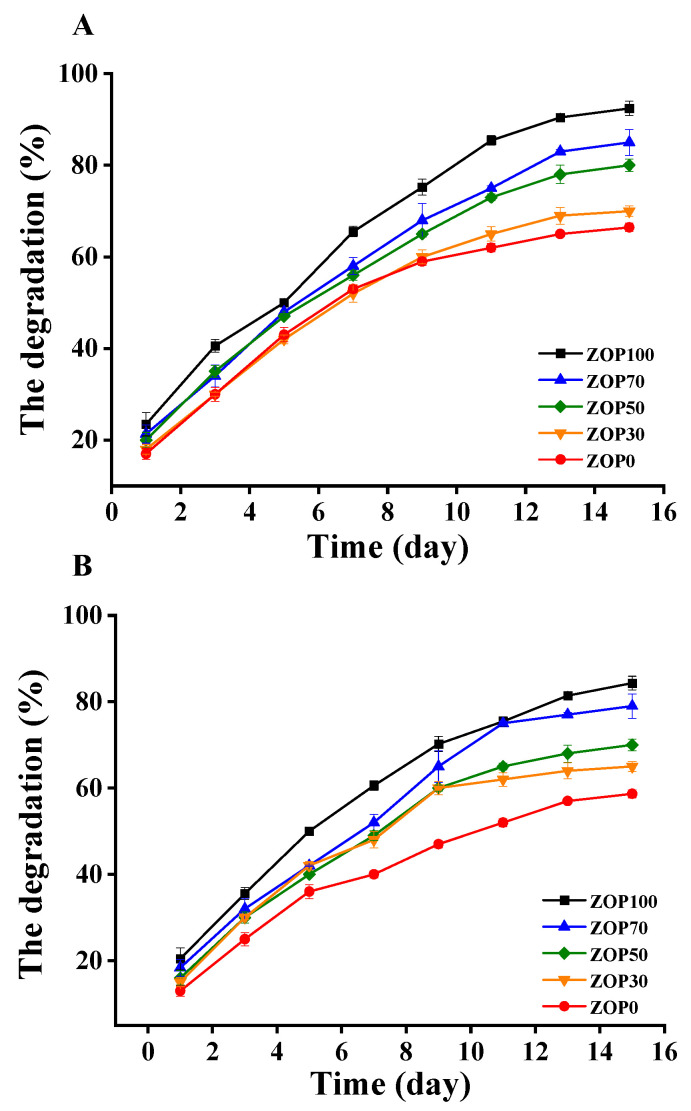
The in vitro degradation profile of different hydrogels in a pH of 1.2 (**A**) and the in vitro degradation profile of different hydrogels in a pH of 7.4 (**B**).

The weight loss of five groups of hydrogels was used to study their degradation in vitro. Previous research data show that, in a PBS solution, the hydrogel network gradually collapses with the destruction of hydrogen bonds [49,50]. In this study, after 15 days of degradation, ZOP0 retained about 66.75 ± 0.85% in a pH of 1.2 (Figure 8A) and 58.67 ± 0.74% in a pH of 7.4 (Figure 8B). Compared with pure CS hydrogel, the mass of ZOP100 under a pH of 1.2 was about 12.45 ± 1.59%, and the mass at a pH of 7.4 was about 19.32 ± 1.40%. In conclusion, the addition of ZOP enhanced the degradation rate of the composite hydrogels. This might be related to the glycosidic bond between monomers [2]. Similar results [4] showed that CS amino protonation accelerated the degradation rate of CS-based hydrogels in an acidic PBS solution. Finally, with the disappearance of hydrogen bonds and the collapse of the hydrogel network, the internal structure was gradually destroyed [13]. 

### 3.13. In Vitro Release

The transport function of hydrogels to molecules was affected by many factors, such as pH, solubility, and pore size [51]. In this study, BSA was selected as the drug-release model to compare the release abilities of hydrogels with different ZOP contents 

**Figure 9:**
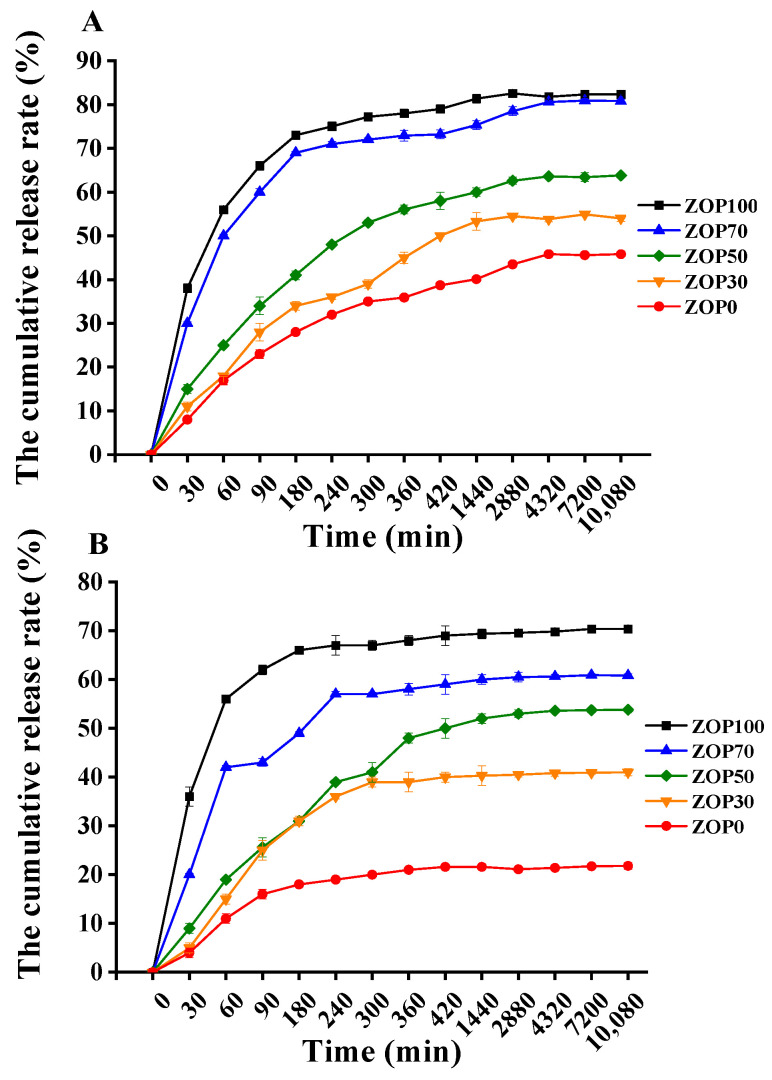
The cumulative release rate (%) of different hydrogels in pH 1.2 PBS (**A**); the cumulative release rate (%) of different hydrogels in pH 7.4 PBS (**B**).

As shown in Figure 9, five samples (ZOP100, ZOP0, ZOP50, ZOP30, and ZOP0) had a faster initial release rate. In fact, the protein on the surface of the hydrogel dissolved easily [31]. After that, the release curve became slow. The BSA release rate of ZOP100 was faster than that of ZOP70, ZOP50, ZOP30, and ZOP0. The release capacity of the hydrogel was found to be consistent with its swelling capacity. Studies have shown that the increase in the swelling rate of the hydrogel leads to larger pores, which is conducive to the transportation of protein molecules from the hydrogel [52]. 

As shown in Figure 9A, in a PBS solution of pH 1.2, ZOP100 released 83.57% BSA after seven days of incubation, while the value for ZOP0 was 45.80 ± 1.65%. As shown in Figure 9B, in a pH of 7.4, the release rate of round hydrogel of ZOP100 within seven days was about 70.35 ± 0.45%, and the release rate of ZOP0 was not more than 22%. Overall, hydrogel-coated BSA was more likely to be released in acidic environments. This might be because the amino group of CS is deprotonated at a high pH, and the network of hydrogels contracts, making it difficult for proteins to be excreted. The internal structure of the hydrogel was almost dense, which was not conducive to the release of proteins [53]. Under acidic conditions, the protonation of the CS amino led to the relaxation of the hydrogel network, so the protein was easy to diffuse into the aqueous gel [54]. In addition, the results of in vitro degradation showed that the hydrogel was more easily degraded in an acidic environment, and the disintegration of the carrier led to the release of BSA. Other studies on hydrogels have shown similar results [4].

### 3.14. Cytotoxicity

**Figure 10:**
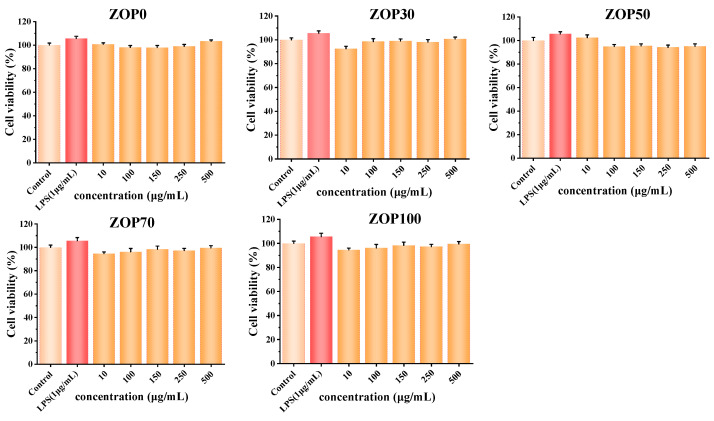
Effect of different proportions of hydrogel on the cell viability of RAW264.7 cells.

In order to evaluate the feasibility of the ZOP/CS hydrogel as a protein carrier, the effect of the hydrogel on the viability of RAW264.7 cells was determined using the CCK-8 method. As shown in Figure 10, compared with the control group, the cell viability of the five groups of samples was more than 90% after 24 h incubation, indicating that the hydrogel had no significant cytotoxic effect on RAW264.7 cells [55].

## 4. Conclusions

In this study, a pH-sensitive complex hydrogel for protein release was prepared. The results showed that the swelling, structural behavior, and degradation mode of the hydrogel mainly depended on the amount of ZOP. The results of the swelling study showed that the hydrogel was sensitive to pH and ionic strength. In addition, in different pH values (1.2 and 7.4), the in vitro biodegradability of the hydrogels was improved by increasing the ZOP content. The protein release of the ZOP/CS hydrogel was investigated using BSA as a model drug. The data showed that the release rate was affected by the ZOP dosage and pH value. The release rate may be affected by the dosage of ZOP and the pH value. The results showed that the hydrogel could be used as a potential pH-sensitive oral protein carrier. In addition, due to its suitable structure, expansion, and texture behavior, the application of ZOP-based hydrogels in tissue engineering seems to be a promising area for investigation.

## Figures and Tables

**Table 1:** Dynamic rheological parameters, gel hardness, and gel springiness of the samples.

Samples	k (Pa s^n^)	n	*R^2^*	Gel Hardness (g)	Gel Springiness (g)
ZOP0	0.189 ± 0.012 ^a^	0.025 ± 0.004 ^a^	0.991	500.497 ± 4.738 ^e^	0.761 ± 0.005 ^a^
ZOP30	0.170 ± 0.034 ^b^	0.017 ± 0.007 ^b^	0.991	521.803 ± 7.198 ^d^	0.692 ± 0.022 ^b^
ZOP50	0.152 ± 0.008 ^c^	0.013 ± 0.005 ^c^	0.990	543.696 ± 2.112 ^c^	0.653 ± 0.096 ^c^
ZOP70	0.136 ± 0.011 ^d^	0.024 ± 0.001 ^a^	0.995	576.965 ± 9.695 ^b^	0.582 ± 0.039 ^d^
ZOP100	0.112 ± 0.015 ^e^	0.012 ± 0.006 ^c^	0.991	606.409 ± 3.265 ^a^	0.550 ± 0.009 ^e^

Results are shown as mean values ± SD in triplicate. Different letters in the same column show a significant difference at *p* < 0.05. “*n*” and “k” are constant values, R^2^ is the determination coefficient.

## Data Availability

The data that support the findings of this study are available within the manuscript.
